# Triamcinolone Modulates Chondrocyte Biomechanics and Calcium-Dependent Mechanosensitivity

**DOI:** 10.3390/ijms27021055

**Published:** 2026-01-21

**Authors:** Chen Liang, Sina Jud, Sandra Frantz, Rosa Riester, Marina Danalache, Felix Umrath

**Affiliations:** 1 Department of Orthopedic Surgery, University Hospital of Tuebingen, 72076 Tuebingen, Germany; 2 Department of Oral and Maxillofacial Surgery, University Hospital of Tuebingen, 72076 Tuebingen, Germany

**Keywords:** glucocorticoids, triamcinolone acetonide, osteoarthritis, chondrocytes, cytoskeleton, mechanotransduction

## Abstract

Glucocorticoids are widely applied intra-articularly to alleviate inflammation and pain in osteoarthritis (OA). However, repeated administration and high local concentrations can lead to crystal deposition on the cartilage surface, contributing to chondrocyte damage and extracellular matrix (ECM) degradation, potentially accelerating OA progression. Calcium-dependent mechanosensors play a critical role in mediating catabolic responses in chondrocytes, but it remains unclear whether glucocorticoids affect chondrocyte mechanosensitivity or biomechanical properties. This in vitro study examined the dose-dependent effects of triamcinolone acetonide (TA) on chondrocyte biomechanics and mechanosensitivity. Primary human chondrocytes (N = 23) were cultured for one week with TA (2 µM–2 mM) or control medium. Cytoskeletal organization was visualized by F-actin staining (N = 6), and cellular elasticity (N = 5) was quantified via atomic force microscopy (AFM). Mechanotransduction was analyzed by Ca^2+^ imaging (Fluo-4 AM) upon AFM-based indentation (500 nN). Expression of matrix-related and mechanosensitive genes (N = 9) was assessed by qPCR. TA exposure induced a concentration-dependent reorganization of the F-actin cytoskeleton, pronounced at 0.2 mM, accompanied by a significant increase in the elastic modulus (*p* < 0.001). TA further augmented Ca^2+^ fluorescence intensity under basal conditions and during mechanical stimulation. Blocking cationic mechanosensitive channels with GsMtx4 (N = 3) markedly reduced the TA-evoked Ca^2+^ influx (*p* < 0.0001). Significant reduction in MMP1 was observed on the transcriptional level (N = 9) after TA-treatment (*p* < 0.05). In summary, TA enhances chondrocyte stiffness through cytoskeletal condensation and amplifies Ca^2+^-dependent mechanotransduction but reduces MMP1 expression, indicating a dual biomechanical response of chondrocytes to OA under exposure of potent corticosteroid.

## 1. Introduction

Osteoarthritis (OA) represents the most common form of chronic articular disease, with an increasing prevalence linked to aging demographics and the rising incidence of obesity [1]. It is characterized by the progressive deterioration of articular cartilage and chronic pain, which leads to significant disability, functional limitations, and considerable economic costs. The etiology of OA is multifactorial, encompassing genetic, biomechanical, and environmental factors, contributing to its complexity and challenges in its management and treatment. Given the current absence of curative therapy, treatment paradigms are largely aiming at symptom alleviation and deceleration of disease progression through either conservative pharmacologic regimens or surgical interventions. Among conservative options, systemic medications frequently suffer from limited joint-specific efficacy and carry the risk of systemic adverse effects [2,3]. In contrast, intra-articular (IA) delivery of therapeutics such as glucocorticoids (GCs) offers a targeted approach, directly modulating local inflammation and cartilage integrity [4]. However, the chondroprotective [5,6] versus chondrotoxic effects [7] of GCs remain paradoxical and context-dependent, modulated by variables including drug concentration, exposure duration, and molecular structure of the specific GC compound. For example, dexamethasone can preserve the extracellular matrix (ECM) in mesenchymal stem cells through the upregulation of metalloprotease inhibitors [8,9]; conversely, it can also alter ECM elasticity by changing its collagen fingerprint [10,11]. Moreover, repeated or high-dose dexamethasone can adversely impact both the ECM composition [9] and cellular functions, leading to oxidative stress, crystal formation, and apoptosis [12,13]. Among clinically available intra-articularly administered GCs, triamcinolone acetonide (TA) appears to offer equal or superior efficacy in terms of pain relief and functional outcomes, when compared to other types of GC, such as methylprednisolone, betamethasone, hydrocortisone, and dexamethasone [14,15]. Clinically, the typical TA dosing recommendations in most common guidelines are 10 mg for small joints (i.e., fingers), 20 mg for medium joints (shoulder and elbow), 20 to 40 mg for large joints (hips and knees), and up to 80 mg for severe cases [16]. However, the optimal dosing strategy for TA to achieve effective anti-inflammatory outcomes while avoiding potential cartilage destruction and cellular damage [15,17,18] remains debatable. Additionally, despite the known wide-ranging physiological effects of GCs, the detailed mechanisms behind many of these actions are still not fully elucidated [19].

Mechanosensing is essential for preserving the health and functionality of musculoskeletal tissues such as cartilage. Cells of load-bearing tissues experience diverse physical stimuli and sustain tissue homeostasis and functionality through reciprocal interactions. Mechanosensitive calcium receptors, especially PIEZO1, can initiate Hippo [20] and nuclear factor-kappaB (NFκB)-signaling [21] and induce programmed cell deaths of chondrocytes [22] with a distinct association to pro-inflammatory cytokines like Interleukin-1 [23]. Other mechanosensitive calcium receptors, like PIEZO2, play a synergistic role with PIEZO1 [24], while TRPV4 (transient receptor potential vanilloid 4) also has an apoptotic effect upon excessive loading [25].

GCs have been shown to induce alterations in cellular and matrix biomechanics, particularly affecting actin cytoskeletal organization, cell adhesion, and cellular mechanosensors [26], all of which play critical roles in maintaining homeostatic dynamics. Another critical area of interest is the role of GCs in maintaining the cellular calcium balance, a process crucial for regulating various cellular functions, including proliferation and programmed cell death [27]. However, whether and to what extent GCs affect cellular mechanics, mechanotransduction, and the underlying mechanisms remain unknown. This study aimed to elucidate the dose-dependent effects of TA on the mechanical properties of chondrocytes and their mechanosensing capabilities. Specifically, we investigated how TA influences the intracellular free calcium (Ca^2+^) concentration upon mechanical stimulation via atomic force microscopy (AFM) and fluorescence microscopy to gain a better understanding of TA-mediated interactions.

## 2. Results

### 2.1. Determination of Optimal TA Concentrations for Effective Experimental Outcomes

**Figure 1:**
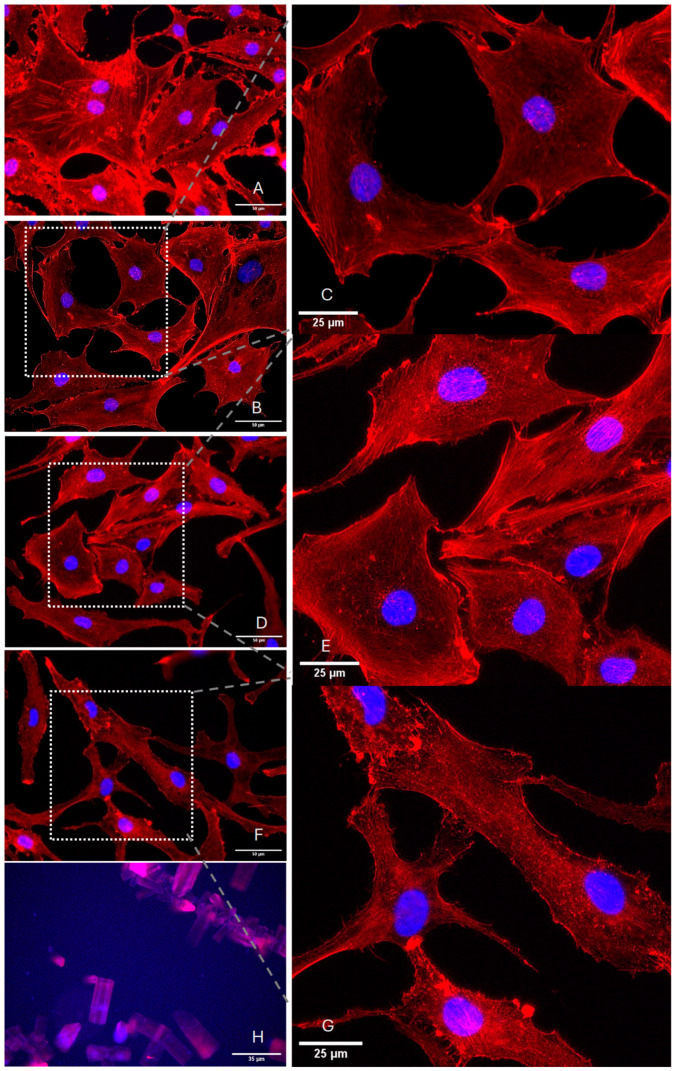
Representative images of chondrocytes incubated with various TA concentrations. After one week of incubation with either TA or the untreated control (DMSO), the isolated chondrocytes were stained for F-actin with phalloidin-AF594 (red), and the cell nuclei were labeled with DAPI (blue) under the following conditions: DMSO as control group ((**A**)—40× magnification); 2 µM TA ((**B**)—40×), ((**C**)—image section in 60×); 20 µM TA ((**D**)—40×), ((**E**)—image section in 60×); 0.2 mM TA ((**F**)—40×), ((**G**)—image section in 60×); and 2 mM TA ((**H**)—20×), where only TA crystals were visible with disrupted nuclei indicated by DAPI staining. The scale bar represents 50 μm for 40× magnification, 25 μm for image sections or 20 μm for 20× magnification. Image sections were cropped in ImageJ software (v1.54r, NIH, Bethesda, MD, USA) and magnified to visualize F-actin activity, which re-organization and condensation are pronounced between 20 µM TA (**E**) and 0.2 mM TA (**G**).

Owing to the solubility limitations of TA, in vitro concentrations are unable to achieve the levels used for intra-articular injections. Therefore, it is necessary to determine a suitable concentration for in vitro experiments (Figure 1A–H). Across the four tested TA concentrations (2 µM, 20 µM, 0.2 mM, and 2 mM), TA incubation induced differential effects in F-actin fiber alignment and ramification. At lower concentrations (2 µM and 20 µM), the chondrocytes exhibited well-defined F-actin stress fibers and characteristic cell morphology with no notable structural differences (Figure 1A–E). At intermediate concentrations (0.2 mM), chondrocytes exhibited slight alterations in F-actin organization (Figure 1F). Particularly at 60× magnification, the distribution of F-actin fibers appeared more condensed and more elongated, with a reduced number of thick actin bundles than at any lower concentration (Figure 1G), whereas the DAPI staining pattern remained the same as the nuclei remained intact. However, at the highest concentration (2 mM), the high abundance of TA crystals severely disrupted F-actin distribution and overall cellular integrity, rendering individual cellular structures indistinguishable (Figure 1H). Since incubation with TA concentrations above 0.2 mM resulted in noticeable macroscopic crystallization, we selected 0.2 mM TA as the effective concentration for further experiments.

### 2.2. TA Alters the Biomechanical Characteristics of Chondrocytes

**Figure 2:**
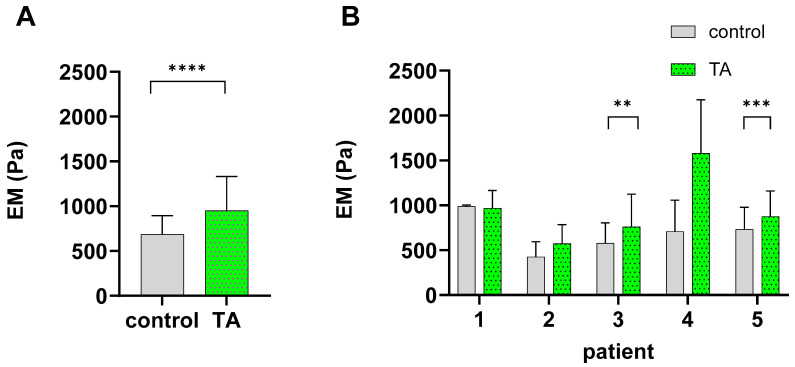
Cellular elasticity of TA-treated chondrocytes. Chondrocytes from 5 patients (30 cells per patient and condition) were treated with either DMSO (control) or 0.2 mM TA for 7 days, and cellular stiffness (elastic modulus, EM) was measured via atomic force microscopy. (**A**) Significantly higher mean EM across all tested patients was observed in the TA group than in the control group (Wilcoxon test, **** *p* < 0.0001). (**B**) Overall, 2 of 5 patients in the single-patient data presented a significantly greater mean EM in the TA group (Wilcoxon test, ** *p* < 0.01; *** *p* < 0.001).

To assess the effect of TA on cellular elasticity, we used atomic force microscopy on treated and untreated chondrocytes to evaluate the cellular elastic modulus (EM). The mean EM value across all five tested patients was 952.3 Pa (SD ± 531.0 Pa, median = 795.5 Pa) for TA-pretreated chondrocytes, whereas the control group presented a mean EM value of 687.2 Pa (SD ± 401.2 Pa, median= 628.0 Pa). Wilcoxon comparison revealed a significant increase (*p* < 0.0001) in the mean EM of 38.6% following TA treatment (Figure 2A). When the data for each of the five tested patients were analyzed individually (Figure 2B), a significantly higher mean EM was observed in 2 out of the 5 patients in the TA-treated group (*p* < 0.001 and *p* < 0.01). This finding supports the original observation of an overall trend toward increasing EM across all measured patients in the TA group.

### 2.3. TA Treatment Significantly Alters Ca^2+^ Dynamics During Mechanical AFM Stimulation

Under AFM indentation, a characteristic fluorescence curve with two peaks and a slow decreasing (T2 and T5, see Section 4.4) plateau can be observed in fluo4-stained chondrocytes. Data from 15% of cells under TA treatment and 7% under control conditions presented no T2-peak, whereas 27% of the measurements in the TA group and 14% in the control group exhibited no T5-peak. Thus, we assumed the presence of T2- and T5-peaks as a distinct response to mechanical stimulation.

**Figure 3:**
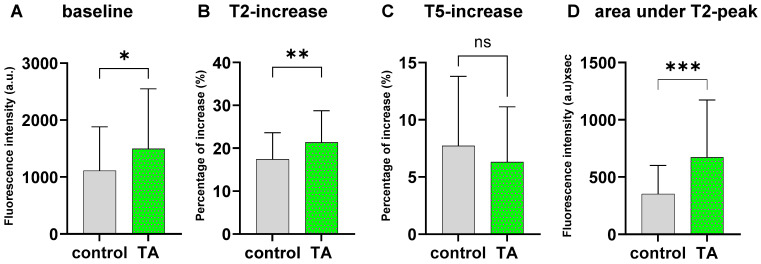
Fluorescence analysis of fluo4-stained intracellular calcium under mechanical stimulation of TA-treated chondrocytes and control for one week in arbitrary units (a.u.). Intracellular fluorescence was recorded for N = 6 donors with n = 10 cells per condition and donor, and the Mann–Whitney U test was performed to detect statistical differences. (**A**) Compared with the control group, the TA group presented a significantly greater baseline fluorescence intensity (* *p* < 0.05). (**B**) The percentual T2-increase during AFM indentation was significantly higher in the TA group (** *p* < 0.01). (**C**) Percentual T5-increase shows no group difference (ns) according to the Mann–Whitney U test (*p* = 0.133). (**D**) Comparison of the mean area under the T2-peak revealed significantly higher values in the TA group (*** *p* < 0.001).

Since cellular stiffness induces Ca^2+^ dynamics [28,29,30], it is expected that TA might modify the baseline Ca^2+^ levels in chondrocytes, which was calculated by subtraction of background fluorescence from the absolute fluorescence intensity of chondrocytes before stimulation (Tstart). Confirming this, our results revealed a significantly enhanced baseline Ca^2+^ intensity in the TA-treated group (*p* < 0.05) before mechanical stimulation, suggesting an enhanced intracellular Ca^2+^ influx as a result of TA treatment (Figure 3A). To eliminate variations arising from baseline differences, the analysis focused on relative Ca^2+^ fluorescence changes, specifically T2- and T5-increase, as indicators of cellular responses to mechanical pressure change at cantilever–chondrocyte contact or cantilever retraction. T2-increase was determined as the difference in fluorescence intensity before stimulation (T1) and the maximum fluorescence intensity during stimulation (T2-peak), relative to T1. Our analysis revealed that TA treatment resulted in a significantly greater percentual increase from T1 to T2 (*p* < 0.0001) compared to the untreated control (Figure 3B). Upon retraction, a second peak (T5) was observed. The height of this peak (T5-increase) was defined as the difference in fluorescence intensity before retraction of the cantilever (T4) until the maximum (T5), relative to the fluorescence intensity at T4. However, a comparative analysis of the percentual T5-increase revealed no significant differences between the TA and control groups (Figure 3C). To evaluate Ca^2+^ turnover during the indentation process, the area under the fluorescence intensity curve of the T2-peak was calculated. In contrast, as indicated by the area under the T2-peak, the impact of TA on Ca^2+^ influx over time during cantilever indentation was significantly greater in the TA group (*p* < 0.001) than in the control group (Figure 3D). This suggests a pronounced increase in Ca^2+^ turnover during the loading phase under TA conditions.

### 2.4. Inhibition of Mechano-Calcium Receptor by GsMtx4

**Figure 4:**
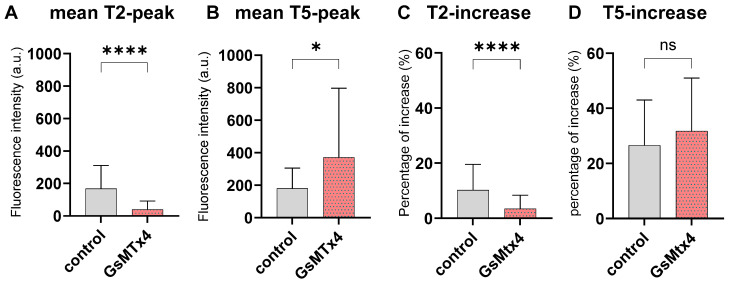
Dynamics of Ca^2+^ in response to mechanical stimulation with and without inhibition of PIEZO channels using GsMTx4. Intracellular fluorescence was recorded for N = 3 donors with n = 10 cells per condition and donor. Mean + SD values of the GsMTx4 group and control were compared using Mann–Whitney U tests (* *p* < 0.05, **** *p* < 0.0001). (**A**) Treatment with GsMTx4 significantly reduced the mean absolute fluorescence intensity of the T2-peak (**** *p* < 0.0001), but (**B**) increased the mean fluorescence intensity of the T5-peak (* *p* < 0.05). (**C**) A significant reduction in the T2-increase was observed in the presence of GsMTx4 (**** *p* < 0.0001). (**D**) Conversely, no significant (ns) difference in T5-increase was detected between the GsMTx4 group and the control group (*p* = 0.288).

GsMtx4 treatment elicited a behavior similar to that of the untreated control, including the presence of both T2- and T5-peaks. Baseline comparisons detected no significant difference between the inhibitor group and the control group (*p* = 0.638) before stimulation, so we added absolute values to this analysis. Notably, a significantly reduced intensity of the T2-peak (Figure 4A, *p* < 0.0001) and increased fluorescence intensity of the T5-peak (Figure 4B, *p* < 0.05) were observed in the GsMtx4 group. Consistent with the results of the TA experiments, we evaluated T2- and T5-increases in the presence of GsMTx4 to investigate Ca^2+^ dynamics during mechanical stimulation. While the T5-increase remained statistically unchanged (Figure 4D), we observed a strikingly significant reduction in the T2-increase (*p* < 0.0001) with GsMTx4 (Figure 4C), which markedly contrasts with the effects observed with TA administration (Figure 3B).

### 2.5. Effect of TA on the Expression of the Tested Genes in Chondrocytes

**Figure 5:**
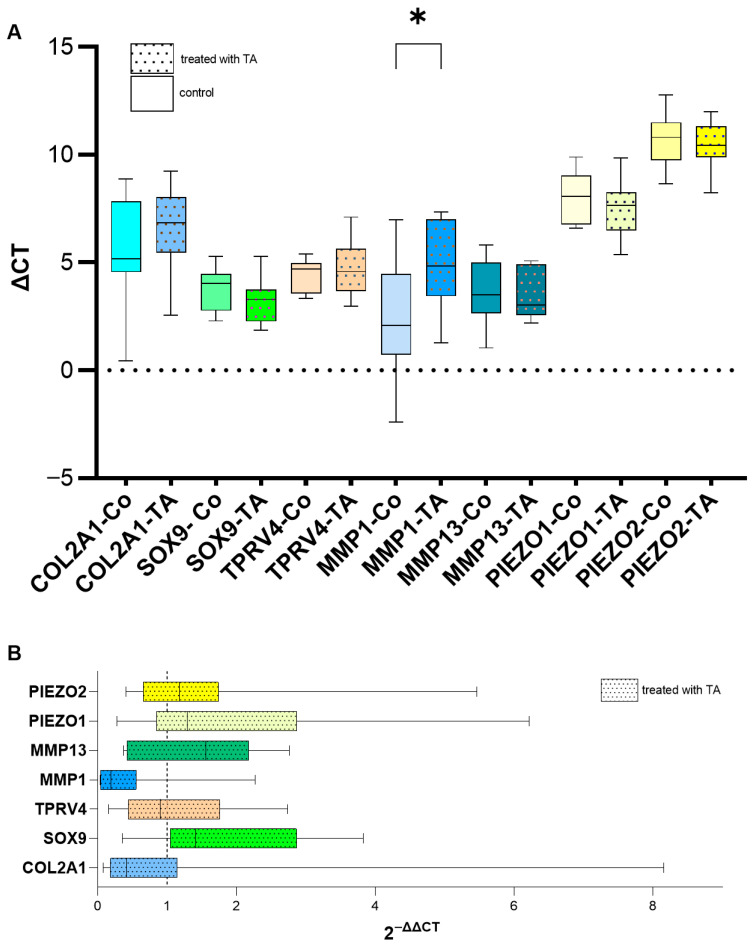
Effect of TA on the gene expression of chondrocytes. Gene expressions of COL2A1, SOX9, TRPV4, PIEZO1, PIEZO2, MMP1, and MMP13 were analyzed by qPCR in chondrocytes derived from N = 9 patients. (**A**) Al-tested conditions after TA and control treatment were displayed in ΔCT values. One-way ANOVA tests with Sidak’s multiple comparisons resulted in highly significant differences between the TA group and untreated controls (*p* < 0.0001) and in a significant reduction in MMP1 expression following TA treatment (* *p* < 0.05), whereas statistics on other conditions did not reach significant levels. (**B**) Relative gene expression is presented as fold change (2^−ΔΔCT^), with untreated controls distributed around 1 after normalization to the housekeeping gene GAPDH. TA treatment showed a clear trend towards reduced COL2A1 and MMP1 expression, whereas mean SOX9 and PIEZO1 expression appeared increased with higher standard deviation.

To delve deeper into the molecular effects of TA, qPCR was utilized to profile the expression of genes associated with key mechanically activated ion channels, namely PIEZO1, PIEZO2, and TRPV4, which play critical roles in articular cartilage. We also examined the expression of SOX9, an important transcription factor, alongside the ECM regulators COL2A1, MMP1, and MMP13. A significant downregulation in MMP1 expression (*p* = 0.013) in the TA group was noted. No significant changes were observed in the expression of COL2A1, SOX9, TRPV4, PIEZO1 and 2, or MMP13 between the TA-treated and untreated group (Figure 5A). Although not statistically significant, an overall trend toward increased expression of PIEZO1 (mean fold change = 2.040 ± SD 1.815) and SOX9 (mean fold change = 1.790 ± SD 1.138), as well as reduced COL2A1 expression (mean fold change = 1.352 ± SD 2.588), was observed, accompanied by substantial inter-individual variability.

## 3. Discussion

Articular cartilage is a prime example of mechanoadaptation, dynamically altering its morphology and composition in response to mechanical stimuli [31,32]. Physiological stress at the cellular level increases expression of ECM components while decreasing the expression of cartilage-degrading matrix metalloproteinases and proinflammatory factors [33,34]. Disruptions in cartilage balance can result from both excessive and insufficient loading, such as from spinal cord injury [35]. These findings underscore the critical role of mechanosensing in maintaining cartilage health.

Glucocorticoids, including TA, are commonly utilized in the management of joint diseases and are typically administered through high-dose intra-articular injections [36]. However, there have recently been concerns about the potential adverse effects of this therapy, as GCs can induce apoptosis and alter the expression of ECM-related genes in chondrocytes and other cell types [37,38], which can result in acceleration of OA onset [39]. Despite their widespread clinical use, the mechanistic basis by which glucocorticoids alter chondrocyte biomechanical behavior remains poorly characterized. Our results showed a distinct dose-dependent response to triamcinolone acetonide (TA), with 0.2 mM eliciting the most pronounced cellular effects. This concentration corresponds to an estimated intra-articular exposure of approximately 1.13 mg TA at an average synovial fluid volume of 13 mL [40], representing a physiologically relevant, yet subclinical range compared to typical therapeutic doses of 20–40 mg. At this concentration, phalloidin-based F-actin staining demonstrated significant condensation of the chondrocyte cytoskeleton, indicative of cytoskeletal remodeling and potential impairment of mechanotransductive functions. Notably, nuclear morphology remained unaffected, suggesting sublethal stress without overt apoptosis. These results are consistent with prior observations by Porter et al., who reported minimal negative effect on viability, proliferation, and anabolic activity, but significant inhibition of catabolic activity, when exposed to 1 nM to 0.2 mM, supporting our choice of 0.2 mM TA as probably the most effective dosage with the least damage [41]. Porter et al. also declared 1 nM as a safe concentration, with no loss of glycosaminoglycan (GAG) at this level. However, as no detectable reorganization of the F-actin cytoskeleton was observed at concentrations below 0.2 mM in our study, it can be assumed that lower concentrations are insufficient to elicit a measurable cellular response relevant to OA-associated inflammation. Supportively, our PCR data already did not show any significant impact on IL-1, -6, or TNF-α at 0.2 mM. Similarly to the study of Porter et al., higher concentrations than 2 mM of TA in our study were also limited by solubility. Notably, already at this concentration, extensive cell death accompanied by cellular detachment and TA crystallization was observed. Euppayo et al. [42] also demonstrated that the application of 2 mM TA for one week clearly had a toxic effect on chondrocytes, as evidenced by the extensive loss of cell structure and TA crystallization, equating to an intra-articular concentration of approximately 11.30 mg. This concentration is even lower than in the findings of Suntiparpluacha et al., who reported significant oxidative stress and diminished chondrocyte viability with any dose higher than 5 mg/mL TA [17]. Conversely, Spitzer et al. demonstrated that administering 20 to 40 mg of TA intra-articularly is safe for larger joints, such as the human knee [43]. As the knee joint is largely composed of perichondrium and articular cartilage with a dense ECM, and because crystallization effects are primarily observed in isolated 2D chondrocytes treated with TA, it can be inferred that in vivo, the perichondrium and ECM provide a protective effect against TA-induced toxicity.

While cartilage stiffness reliably decreases in early OA due to ECM degradation, the stiffness at a cellular level (micro) exhibits variable trends influenced by measurement techniques, tissue zonal origin, and disease severity [44]. Nevertheless, investigating chondrocyte biomechanics remains crucial, as alterations in cellular stiffness may reflect early mechanobiological changes that precede matrix-level degeneration [45]. Despite this variability at the cellular level, cartilage matrix stiffness is widely recognized as a reliable early biomarker of OA, primarily reflecting the progressive disruption of the collagen–proteoglycan network [46,47].

It is known that reduced mechanical properties of the ECM and pericellular matrix (PCM) are markers of OA onset and progression [46] and that increased matrix stiffness drives stress fiber formation in the actin-based cytoskeleton [48]; thus, it can be assumed that these effects shape the biomechanical profile of chondrocytes and implicitly influence their mechanosensing abilities. Therefore, we examined how this modulation impacts the biomechanical signatures of single cells and their calcium profiles in response to mechanical forces. Our results demonstrated a notable increase in cellular EM following TA treatment. Consistently, Trickey et al. reported a mean instantaneous modulus of 630 (±500 Pa) in untreated osteoarthritic chondrocytes, which aligns closely with our control group’s EM measurement of 687.2 (±401.2 Pa) [49]. Similarly, Fackler et al. observed a significantly reduced aggregate modulus in articular cartilage explants following the application of methylprednisolone and TA [50]. These findings, which align with our data, underscore the dual nature of TA effects, influencing biomechanical properties from the ECM and PCM levels down to the single-cell level. While Fackler et al. did not detect notable changes in the biochemical composition of the ECM, and previous studies have documented a significant stepwise reduction in the EM of both the ECM and the PCM during cartilage degeneration [51,52], it is conceivable that the TA-induced stiffening of chondrocytes may be linked to a softening effect on the PCM.

Given that the cytoskeleton functions as a link between chondrocytes, the PCM, and the ECM and that mechanical signals can prompt the assembly of focal adhesion complexes on chondrocytes, thus reorganizing the cytoskeleton [53], it is essential to investigate global Ca^2+^ dynamics as indicators of mechanosensitivity. Our AFM stimulation, coupled with fluo-4 labeling, revealed an elevated Ca^2+^ profile in cells treated with TA prior to mechanical stimulation. Additionally, we observed a characteristic fluorescence signal with two distinct peaks separated by a descending plateau phase throughout the indentation procedure. The initial peak (T2) reflects the reaction of the cell to the pressure applied at the cantilever–chondrocyte interface, indicating rapid Ca^2+^ influx. Such kind of Ca^2+^ surge is likely mediated by mechanosensitive receptors such as PIEZO1, PIEZO2, and TRPV4 [24,54,55], with PIEZO1 and PIEZO2 playing pivotal roles in compression sensitivity [56]. The rapid decline following the T2-peak can be attributed to a restoring force arising from cellular viscoelasticity [57]. The plateau phase not only reflects the deformation of the cell through sustained compression but also indicates that the cell membrane remained intact during indentation. The gradual decline in the plateau phase can be attributed to photobleaching effects [58]. In contrast, the second peak is initially accompanied by a calcium increase due to the cell membrane adhesion force during detachment [59] and is then characterized by a rapid decrease, which is correlated with cell relaxation following cantilever retraction.

To verify that these two peaks accurately represent responses from mechanically activated calcium channels, we employed GsMtx4, a selective inhibitor known to target cationic mechanosensitive channels associated with the PIEZO and TRP families [60]. We observed a marked reduction in the T2-peak intensity and an increase in T5-peak intensity with GsMtx4 treatment. These results highlight the T2- and T5-peaks as distinct indicators of calcium-dependent mechanotransduction in chondrocytes. Similarly, Shen et al. observed a characteristic curve following AFM indentation that varied with cantilever position and applied force. This curve also included an approach and retraction phase with minimal force change, interspersed with a period of significant force increase or decrease [30]. Taken together, these results suggest that mechanosensitive ion channels require only a small stimulus to activate and particularly react to either positive or negative changes in force instead of constant pressure.

Regarding the impact of TA on chondrocyte calcium dynamics, our findings revealed a markedly greater response of TA-treated chondrocytes during the mechanical stimulation loading phase (T2-increase), with no significant response observed during cantilever retraction (T5-increase). This finding indicates that TA amplifies mechanosensitivity in chondrocytes in the loading phase of our AFM pressure (500 nN), which is known to be primarily mediated by PIEZO1, whereas other channels, such as PIEZO2, serve at moderate strain [56]. Interestingly, GsMtx4 had the opposite effect on T2-increase compared to TA, suggesting that TA might amplify the activity of Ca^2+^-dependent mechanosensitive channels such as PIEZO channels, which is limitedly supported by our results at the gene expression level.

Elevated intracellular Ca^2+^ concentrations are commonly associated with impaired function and reduced viability of chondrocytes [61]. In this study, treatment with 0.2 mM TA resulted in increased baseline Ca^2+^ fluorescence, indicating enhanced intracellular calcium levels. A trend toward increased PIEZO1 expression was observed following TA exposure, while PIEZO2 and TRPV4 expression remained unchanged; however, these differences did not reach statistical significance. The absence of significant effects may be attributed to the limited sample size (N = 9) and donor variability. The observed TA-induced elevation in intracellular Ca^2+^ levels, alongside relatively stable expression levels of mechanically activated ion channels such as PIEZO1, PIEZO2, and TRPV4, may indicate functional modulation of mechanotransduction rather than transcriptional reprogramming. This suggests that TA exerts a transient influence on mechanosensitive signaling pathways at the ionic or post-translational level, without initiating sustainable gene expression changes. Such transient modulation may be relevant for maintaining or restoring chondrocyte functional integrity under mechanically or chemically induced stress conditions.

From a gene perspective, SOX-9 and COL2A1 work as essential cellular and intercellular framework components, which are usually downregulated in OA chondrocytes [62,63]. Euppayo et al. [42] also demonstrated that treatment with TA can result in a significant downregulation of COL2A1 mRNA expression and a concomitant reduction in GAG synthesis in canine articular cartilage explants. In human chondrocytes, our study results showed a similar trend in COL2A1 reduction. Interestingly, as SOX-9 is usually a positive regulator of COL2A1 [64], we observed increasing expression of SOX-9 in opposite to the trend of COL2A1. Consistently, increased expression of SOX-9 can be referred to exposure to TA [65]. In summary, these results hint that TA can probably positively influence the same regulatory mechanism of SOX-9 transcription, which also initiates OA [63]. Although overexpression of SOX-9 has been associated with suppression of COL2A1 under certain differentiation states of chondrocytes [66], neither change in our study reached statistical significance, limiting definitive conclusions regarding their regulatory interplay.

Il-1α, -β, -6, and TNF-α are known pro-inflammatory cytokines, which can be modulated by TA systemically [67]. Intra-articular application of TA at a similar dosage can provide protective effects on cartilage, specifically decelerated loss of GAGs, when chondrocytes are exposed to inflammatory cytokines such as Il-1β [41]. Unexpectedly, intracellular cytokines were not relevantly influenced by TA at the gene level in this study, underlining its distinct activation pathway in OA chondrocytes only after exposure to inflammation.

Inflammation can promote the synthesis and overexpression of MMPs. Specifically, a combined increase in Il-1β and TNF-α can stimulate the expression of MMP1, 3, and 13 [68], which lead to degradation of collagen II and GAG. MMP1, whose levels in the synovium are negatively correlated with the grade of OA [69], is a collagenase predominantly sourced from the upper layer of the cartilage, while MMP13 is mostly present in chondrocytes [70]. In line with previous reports [9,71], low-dose corticosteroids seem to suppress MMP-driven cartilage degradation. However, in contrast to findings derived from animal models, our results suggest that TA modulates MMP1 in human chondrocytes, with no significant effect observed on MMP13 expression. These findings highlight the need for further investigation into the molecular effects of TA on MMP activity in the human OA synovium and synovial cell populations.

Regrading study limitations, it is important to mention that although the articular cartilage used in this study was obtained from macroscopically healthy tissue, the samples were sourced from OA patients with higher grades. This limiting factor may have influenced the observed values. Further study using healthy human cartilage could provide more comprehensive understanding of this issue; however, surgical extraction of healthy cartilage is medically and ethically unjustified. Yet, the observed reduced expression of MMP1, but enhanced calcium responsiveness and chondrocyte stiffness, may explain the biological effect as a double-edged sword of TA in the progression of OA.

A key limitation of this study lies in the origin and condition of the cartilage samples. Although the articular cartilage was harvested from macroscopically intact regions, all tissue was obtained from patients with end-stage OA (Kellgren–Lawrence grades III–IV). This pre-existing disease state may have influenced baseline gene expression, cellular responsiveness, and mechanotransductive behavior, potentially confounding the interpretation of TA-specific effects. Additionally, the relatively small sample size (N = 9), along with inter-donor variability, further limits the statistical power of this study. Despite these limitations, the observed TA-induced reduction in MMP1 expression, coupled with increased intracellular calcium responsiveness and altered chondrocyte stiffness, suggests a complex, potentially dualistic effect of TA on cartilage biology. These findings raise the possibility that TA may simultaneously confer protective effects through MMP suppression while also altering mechanosensitive signaling pathways in a manner that could affect long-term chondrocyte function and OA progression. Further studies incorporating larger sample sizes and alternative models of early OA are warranted to better elucidate these mechanisms.

## 4. Materials and Methods

### 4.1. Isolation of Primary Chondrocytes

Articular cartilage samples were obtained from patients (N = 23) undergoing total knee arthroplasty at the Department of Orthopedic Surgery, University Hospital of Tübingen, and Winghofer Medicum Clinic in Rottenburg, Germany. The study cohort included patients with symptomatic OA with at least radiographic Kellgren–Lawrence grade III or IV and operative indication for knee arthroplasty. Collected cartilage was anonymized and subsequently separated from the subchondral bone using a scalpel, washed with phosphate-buffered saline (PBS), and sectioned into small pieces, approximately 4 mm in diameter and 1 mm in thickness. Chondrocyte isolation was performed enzymatically overnight at 37 °C in DMEM (Gibco, Life Technologies, Waltham, MA, USA) supplemented with 10% fetal calf serum (Sigma-Aldrich, St. Louis, MO, USA), 1% penicillin–streptomycin (Sigma-Aldrich, St. Louis, MO, USA), and 1.25% amphotericin B (Sigma-Aldrich, St. Louis, MO, USA), supplemented with 0.5 mg/mL collagenase XI (Sigma-Aldrich) and 0.5 mg/mL dispase (Roche, Basel, Switzerland).

### 4.2. Determination of the Optimal TA Dosage

To assess the optimal concentration of TA (Sigma-Aldrich), isolated chondrocytes were seeded in a 6-well plate (Greiner Bio-One, Frickenhausen, Germany) at a density of 5 × 10^4^ cells and treated for one week with chondrocyte medium containing 1 mL of different TA concentrations (2 µM, 20 µM, 0.2 mM, or 2 mM) dissolved in dimethyl sulfoxide (DMSO). At 2 mM, clear crystallization can be observed macroscopically, so that no higher dose can be dissolved without supplementation of clinically used auxiliary substances such as benzyl-alcohol. A control group, which was treated with DMSO only, was also included. Following TA incubation, the cells were stained with DAPI (Invitrogen, Waltham, MA, USA) and phalloidin-Alexa Fluor 594 (BioLegend, San Diego, CA, USA) to label the nuclei and F-actin, respectively. Images were captured with a Leica DMi8 fluorescence microscope (Leica, Wetzlar, Germany).

### 4.3. Elasticity Measurements—Atomic Force Microscopy

Cellular elasticity was measured using an atomic force microscope (AFM) (CellHesion200 AFM system; Bruker, Billerica, MA, USA), which was mounted onto an inverted light microscope (AxioObserver D1; Carl Zeiss Microscopy, Jena, Germany). AFM measurements were performed exactly as previously described. Briefly, a cantilever with a 5 µm spherical tip (#SAA-SPH-5UM, Bruker, Billerica, MA, USA) was used for cell indentations. Indentation curves were sampled at 2 kHz with a 5 nN force trigger. The cantilever was calibrated on the extended curve, and the spring constant was determined via the thermal noise method integrated into the device software (Bruker). A total of 30 cells were subjected to AFM indentation measurements per condition and donor (N = 5), with each cell being measured three times. The cell stiffness, expressed as the elastic modulus [37], was calculated from the force–distance curves via the Hertz fit model for spherical indenters available in the data processing software (Version 5.0.86, Bruker).

### 4.4. Mechanically Induced Intracellular Calcium Dynamics

To measure the dynamics of intracellular Ca^2+^ concentrations, cartilage samples were incubated in chondrocyte medium supplemented with either of 0.2 mM TA or DMSO as a control. After one week, the chondrocytes were isolated, and 3 × 10^4^ cells per donor (N = 6) were seeded into collagen (0.1% *w*/*v*)-coated Petri dishes (TPP Techno Plastic Products AG, Trasadingen, Switzerland). After adhesion, the cells were washed with a phosphate-buffered saline (HBSS) solution (Sigma-Aldrich) and then stained for 1 h in a Ca^2+^-sensitive dye solution containing 4 µM fluo-4 AM mixed with DMSO and Pluronic F-127 (Invitrogen, Waltham, MA, USA) at 37 °C. To investigate the mechanically induced changes in Ca^2+^ dynamics, the chondrocytes were subjected to AFM indentation using a tipless cantilever (spring constant: 40 N/m, All-In-One-Al-Tipless-tip D; BudgetSensors, Sofia, Bulgaria) with a force of 500 nN and a contact time of 60 s. Intracellular fluorescence during stimulation was recorded (n = 10 cells per condition and donor) using an AxioObserver D1 microscope (Carl Zeiss) equipped with an Axiocam 503 mono camera (Carl Zeiss) and analyzed with ZEN Blue Pro software (v3.3, Carl Zeiss). To evaluate changes in the cellular Ca^2+^ concentration fluorescence during AFM stimulation, a 5 µm region of interest (ROI) was placed on the AFM-indented chondrocyte to capture the fluorescence signal (ROI1). Additionally, fluorescence signals from unstimulated cells (ROI2) and background fluorescence (ROI3) were recorded simultaneously and used for normalization. To assess the baseline fluorescence intensity, the absolute fluorescence intensity in the ROIs was measured before stimulation (Tstart) and corrected for background fluorescence via ROI3.

**Figure 6:**
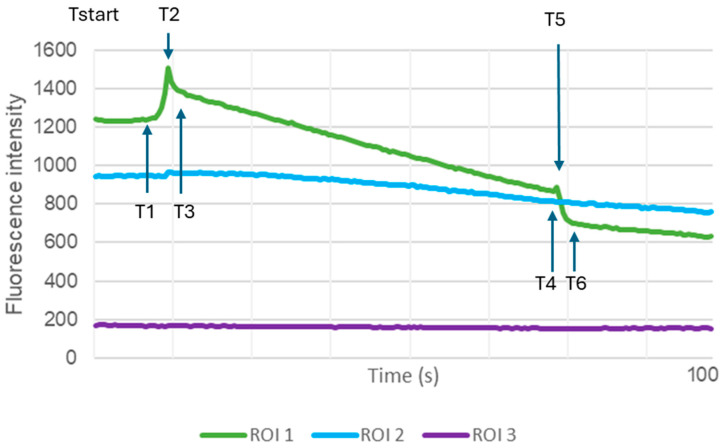
Fluorescence intensity curve by Ca^2+^ imaging with fluo-4: Representative fluorescence intensity (arbitrary units) measurements for ROI1, ROI2 (blue), and ROI3 (purple) during AFM-mediated mechanical stimulation using a tipless cantilever (500 nN and 60 s contact time). The time points on the X-axis are defined as follows: measurement starts at 0 s (Tstart), initiation of cantilever indentation (T1), loading phase between T1 and T2 (T2 increase), peak force of 500 nN (T2), contact time with steady pressure (T3), beginning of cantilever retraction (T4), peak of maximum fluorescence during retraction (T5), and complete cantilever retraction (T6).

When mechanical stimulation was applied, the fluorescence signals revealed a markedly mechanically induced Ca^2+^ peak (Figure 6). Upon first contact of the cantilever with the chondrocyte (T1), an increase in fluorescence intensity (T2-increase) was observed, reaching a peak at the maximum preset indentation force of 500 nN (T2-peak). During the 60 s contact time, the fluorescence signals initially decreased rapidly before stabilizing at a plateau with a modest, sustained decline (T3). The plateau continued throughout the contact time, lasting until cantilever retraction. Upon retraction, a second peak (T5) was observed before a rapid decline in fluorescence until complete cantilever retraction (T6). We defined T2 increase as the percentage increase from T1 to T2 to T1, with T1 serving as the baseline reference, and similarly defined T5 increase. The loss of contact was marked at time point T6. This phenomenon was detectable exclusively in ROI1, which was placed in the exact position of cantilever contact; no T2- or T5-peaks were observed in regions outside the predefined 5 µm diameter.

To evaluate Ca^2+^ turnover during the indentation process, the area under the fluorescence intensity curves of the T2-peak and T5-peak was used as an estimate of Ca^2+^ influx [72,73]. Since the area under the descending plateau can vary considerably, we considered the area under the T2-peak as the primary response of chondrocytes to loading of mechanical pressure. Thus, differences were normalized by interpolating the fluorescence values between T1 and T3. The absolute fluorescence intensities per area were recorded as Y values, and the corresponding time points were recorded as X values, which were incremented regularly to obtain subsequent points, with most measurements taken at a frequency of two readings per second. The slope of the resulting interpolation line between T1 and T3 was calculated accordingly. We employed the trapezoidal rule to calculate the area of individual trapezoids, summing these values to obtain the total area. The area under the T2-peak was determined by subtracting the area beneath the interpolation line over time from the total area under the T2-peak.

### 4.5. Evaluation of Ca^2+^ Dynamics Under GsMTx4 Treatment

To investigate the involvement of mechanosensitive ion channels, cells were treated with GsMTx4 (40 µM, 10 min, HY-P1410, Hycultec, Beutelsbach, Germany) to inhibit PIEZO ion channels. Following GsMTx4 treatment for 1 h at 37 °C, Ca^2+^ measurements were conducted under AFM-mediated mechanical stimulation for N = 3 patients, as described above.

### 4.6. Gene Expression Analysis via qPCR

qPCR was performed analogously to previously published protocol [74,75]. After the chondrocytes were incubated with 0.2 mM TA, total RNA was extracted from the chondrocytes (N = 9) via the NucleoSpin RNA Isolation Kit (Macherey-Nagel, Düren, Germany). The purity and concentration of RNA were determined via a NanoDrop™ (Thermo Scientific, Waltham, MA, USA) spectrophotometer. Subsequently, 1 µg of RNA was reverse-transcribed using innuSCRIPT reverse transcriptase (Analytik Jena, Jena, Germany). A total of 1 µL of cDNA (50 ng) was analyzed in duplicate via quantitative RT–PCR (qRT–PCR) with gene-specific primers and SYBR Select Master Mix for CFX (Life Technologies GmbH). The primer pairs used for TRPV4 (for 5′-TTC ATT AAC TCG CCC TTC CG-3′, rev 5′-GGT TCT CCG TCA GGT AGT TG-3′), PIEZO1 (for 5′-TCT TGG TGG TCT CCT CTGT C-3′, rev 5′-GCA TCC ACA TCC CTC TCA TC-3′), PIEZO2 (for 5′-GAA GCA GAC AAA CAG AAA GCC-3′, rev 5′-TCC TCT TCC TCC TCT TCA CTA TC-3′), MMP1 (for 5′-GAC AGA GAT GAA GTC CGG TTT-3′, rev 5′-GCC AAA GGA GCT GTA GAT GTC-3′), MMP13 (for 5′-AGC CAC TTT ATG CTT CCT GA-3′, rev 5′-TGG CAT CAA GGG ATA AGG AAG-3′), SOX9 (for 5′-CTG GGA AAG CTC TGG AG-3′, rev 5′-CGT TCT TCA CCG ACT TCC TC-3′), and COL2A1 (for 5′-CCT CAA GGA TTT CAA GGC AAT-3′, rev 5′-GTT TTC CAG CTT CAC CAT CAT C-3′) were obtained from IDT Integrated DNA Technologies (Leuven, Belgium). qRT–PCR was performed using a QuantStudio3 system (Thermo Scientific) and analyzed with the device’s integrated data analysis software (Thermo Scientific). Relative expression levels were calculated via the ΔCt (2^−ΔΔCt^) method as previously described [76], with GAPDH serving as the reference gene.

### 4.7. Statistical Analysis

The data are graphically displayed as means with standard deviations (SDs). Data normality was assessed via QQ plots and Kolmogorov–Smirnov tests. Based on normality, statistical comparisons were performed via the Wilcoxon test for cell elasticity and Mann–Whitney U test for Ca^2+^-measurements. For PCR data, one-way ANOVA with Sidak’s multiple comparison analyses were performed due to the normal distribution of ΔCT data. Data analysis and graphical representation were carried out by GraphPad Prism 10.6.1, and statistical significance was determined with an alpha value of 0.05.

## 5. Conclusions

In summary, our study demonstrates that TA exerts a dual and dose-dependent impact on human articular chondrocytes by modulating both their biomechanical properties and mechanosensitivity. At pharmacologically relevant concentrations, TA induces slight cytoskeletal changes, leading to increased cellular stiffness and enhanced Ca^2+^-dependent mechanotransduction. However, on the transcriptional level, TA reduces MMP1 expression but not the expression of pro-inflammatory cytokines, suggesting its distinct chondroprotective characteristic, rather than directly suppressing inflammatory signaling, within the context of osteoarthritic cartilage.

## Data Availability

The data presented in this study are available on request from the corresponding author in an anonymous form due to patient privacy. Traceable patient data are not available due to data protection.

## Abbreviations

AFM: Atomic Force microscopy; GC: glucocorticoids; TA: Triamcinolone; DAPI: 4′,6-diamidino-2-phenylindole; GsMTx4: Grammostola spatulata mechanotoxine 4, M-theraphotoxine-Gr1a, M-TRTX-Gr1a; SOX9: SRY-Box Transcription Factor 9; MMP: metalloproteinase; COL2A1: Collagen Type 2 Alpha 1 Chain; GAPDH: Glyceraldehyde-3-phosphate dehydrogenase; DMSO: Dimethyl sulfoxide; IL-1: Interleukin-1; PCM: pericellular matrix; ECM: extracellular matrix; CT: cycle threshold; GAG: glycosaminoglycan.

## References

[1] Allen K.D., Thoma L.M., Golightly Y.M. (2022). Epidemiology of osteoarthritis. Osteoarthr. Cartil..

[2] Michael J.W., Schlüter-Brust K.U., Eysel P. (2010). The epidemiology, etiology, diagnosis, and treatment of osteoarthritis of the knee. Dtsch. Arztebl. Int..

[3] Kloppenburg M., Berenbaum F. (2020). Osteoarthritis year in review 2019: Epidemiology and therapy. Osteoarthr. Cartil..

[4] Wittenauer R., Smith L., Aden K. (2013). Background Paper 6.12 Osteoarthritis.

[5] Huebner K.D., Shrive N.G., Frank C.B. (2014). Dexamethasone inhibits inflammation and cartilage damage in a new model of post-traumatic osteoarthritis. J. Orthop. Res..

[6] Roach B.L., Kelmendi-Doko A., Balutis E.C., Marra K.G., Ateshian G.A., Hung C.T. (2016). Dexamethasone Release from Within Engineered Cartilage as a Chondroprotective Strategy Against Interleukin-1α. Tissue Eng. Part A.

[7] Dragoo J.L., Danial C.M., Braun H.J., Pouliot M.A., Kim H.J. (2012). The chondrotoxicity of single-dose corticosteroids. Knee Surg. Sports Traumatol. Arthrosc..

[8] Brew K., Nagase H. (2010). The tissue inhibitors of metalloproteinases (TIMPs): An ancient family with structural and functional diversity. Biochim. Biophys. Acta.

[9] Richardson D.W., Dodge G.R. (2003). Dose-dependent effects of corticosteroids on the expression of matrix-related genes in normal and cytokine-treated articular chondrocytes. Inflamm. Res..

[10] Daniel C., Traub F., Sachsenmaier S., Riester R., Mederake M., Konrads C., Danalache M. (2023). An exploratory study of cell stiffness as a mechanical label-free biomarker across multiple musculoskeletal sarcoma cells. BMC Cancer.

[11] Umrath F., Pfeifer A., Cen W., Danalache M., Reinert S., Alexander D., Naros A. (2022). How osteogenic is dexamethasone?—Effect of the corticosteroid on the osteogenesis, extracellular matrix, and secretion of osteoclastogenic factors of jaw periosteum-derived mesenchymal stem/stromal cells. Front. Cell Dev. Biol..

[12] Gordon G.V., Schumacher H.R. (1979). Electron microscopic study of depot corticosteroid crystals with clinical studies after intra-articular injection. J. Rheumatol..

[13] McCarty D.J. (1994). Crystals and arthritis. Dis. Mon..

[14] Garg N., Perry L., Deodhar A. (2014). Intra-articular and soft tissue injections, a systematic review of relative efficacy of various corticosteroids. Clin. Rheumatol..

[15] Wernecke C., Braun H.J., Dragoo J.L. (2015). The Effect of Intra-articular Corticosteroids on Articular Cartilage: A Systematic Review. Orthop. J. Sports Med..

[16] Zentiva Fachinfo Triam Injekt. https://www.zentiva.de/-/media/files/zentivade/produkte/triam-40mg-lichtenstein/_de_fi_triam_40mg_lichtenstein.pdf.

[17] Suntiparpluacha M., Tammachote N., Tammachote R. (2016). Triamcinolone acetonide reduces viability, induces oxidative stress, and alters gene expressions of human chondrocytes. Eur. Rev. Med. Pharmacol. Sci..

[18] Weitoft T., Öberg K. (2019). Dosing of intra-articular triamcinolone hexacetonide for knee synovitis in chronic polyarthritis: A randomized controlled study. Scand. J. Rheumatol..

[19] Barnes P.J. (2011). Glucocorticosteroids: Current and future directions. Br. J. Pharmacol..

[20] Cai X., Warburton C., Perez O.F., Wang Y., Ho L., Finelli C., Ehlen Q.T., Wu C., Rodriguez C.D., Kaplan L. (2024). Hippo-PKCζ-NFκB signaling axis: A druggable modulator of chondrocyte responses to mechanical stress. iScience.

[21] Deng Y., Lu J., Li W., Wu A., Zhang X., Tong W., Ho K.K., Qin L., Song H., Mak K.K. (2018). Reciprocal inhibition of YAP/TAZ and NF-κB regulates osteoarthritic cartilage degradation. Nat. Commun..

[22] Nims R., Palmer D.R., Kassab J., Zhang B., Guilak F. (2024). The chondrocyte “mechanome”: Activation of the mechanosensitive ion channels TRPV4 and PIEZO1 drives unique transcriptional signatures. FASEB J..

[23] Lee W., Nims R.J., Savadipour A., Zhang Q., Leddy H.A., Liu F., McNulty A.L., Chen Y., Guilak F., Liedtke W.B. (2021). Inflammatory signaling sensitizes Piezo1 mechanotransduction in articular chondrocytes as a pathogenic feed-forward mechanism in osteoarthritis. Proc. Natl. Acad. Sci. USA.

[24] Lee W., Leddy H.A., Chen Y., Lee S.H., Zelenski N.A., McNulty A.L., Wu J., Beicker K.N., Coles J., Zauscher S. (2014). Synergy between Piezo1 and Piezo2 channels confers high-strain mechanosensitivity to articular cartilage. Proc. Natl. Acad. Sci. USA.

[25] Xu B., Xing R., Huang Z., Yin S., Li X., Zhang L., Ding L., Wang P. (2019). Excessive mechanical stress induces chondrocyte apoptosis through TRPV4 in an anterior cruciate ligament-transected rat osteoarthritis model. Life Sci..

[26] Zhu H., Li J., Li Y., Zheng Z., Guan H., Wang H., Tao K., Liu J., Wang Y., Zhang W. (2021). Glucocorticoid counteracts cellular mechanoresponses by LINC01569-dependent glucocorticoid receptor-mediated mRNA decay. Sci. Adv..

[27] Giorgi C., Danese A., Missiroli S., Patergnani S., Pinton P. (2018). Calcium Dynamics as a Machine for Decoding Signals. Trends Cell Biol..

[28] Du G., Chen W., Li L., Zhang Q. (2022). The potential role of mechanosensitive ion channels in substrate stiffness-regulated Ca^2+^ response in chondrocytes. Connect. Tissue Res..

[29] Yuan G., Xiong Z., Ke X., Wang G., Liu X., Li Z. (2025). Exploring the Multifactorial Regulation of PIEZO1 in Chondrocytes: Mechanisms and Implications. Int. J. Med. Sci..

[30] Shen X., Hu L., Li Z., Wang L., Pang X., Wen C.Y., Tang B. (2021). Extracellular Calcium Ion Concentration Regulates Chondrocyte Elastic Modulus and Adhesion Behavior. Int. J. Mol. Sci..

[31] Vincent T.L., Wann A.K.T. (2019). Mechanoadaptation: Articular cartilage through thick and thin. J. Physiol..

[32] Carter D.R., Beaupré G.S., Wong M., Smith R.L., Andriacchi T.P., Schurman D.J. (2004). The mechanobiology of articular cartilage development and degeneration. Clin. Orthop. Relat. Res..

[33] Wright M.O., Nishida K., Bavington C., Godolphin J.L., Dunne E., Walmsley S., Jobanputra P., Nuki G., Salter D.M. (1997). Hyperpolarisation of cultured human chondrocytes following cyclical pressure-induced strain: Evidence of a role for alpha 5 beta 1 integrin as a chondrocyte mechanoreceptor. J. Orthop. Res..

[34] Nam J., Perera P., Liu J., Wu L.C., Rath B., Butterfield T.A., Agarwal S. (2011). Transcriptome-wide gene regulation by gentle treadmill walking during the progression of monoiodoacetate-induced arthritis. Arthritis Rheum..

[35] Vanwanseele B., Eckstein F., Knecht H., Stüssi E., Spaepen A. (2002). Knee cartilage of spinal cord-injured patients displays progressive thinning in the absence of normal joint loading and movement. Arthritis Rheum..

[36] Popma J.W., Snel F.W., Haagsma C.J., Brummelhuis-Visser P., Oldenhof H.G., van der Palen J., van de Laar M.A. (2015). Comparison of 2 Dosages of Intraarticular Triamcinolone for the Treatment of Knee Arthritis: Results of a 12-week Randomized Controlled Clinical Trial. J. Rheumatol..

[37] Pemmari A., Leppänen T., Hämäläinen M., Moilanen T., Vuolteenaho K., Moilanen E. (2020). Widespread regulation of gene expression by glucocorticoids in chondrocytes from patients with osteoarthritis as determined by RNA-Seq. Arthritis Res. Ther..

[38] Nakazawa F., Matsuno H., Yudoh K., Watanabe Y., Katayama R., Kimura T. (2002). Corticosteroid treatment induces chondrocyte apoptosis in an experimental arthritis model and in chondrocyte cultures. Clin. Exp. Rheumatol..

[39] McAlindon T.E., LaValley M.P., Harvey W.F., Price L.L., Driban J.B., Zhang M., Ward R.J. (2017). Effect of Intra-articular Triamcinolone vs Saline on Knee Cartilage Volume and Pain in Patients with Knee Osteoarthritis: A Randomized Clinical Trial. JAMA.

[40] Li W., Abram F., Pelletier J.P., Raynauld J.P., Dorais M., d’Anjou M.A., Martel-Pelletier J. (2010). Fully automated system for the quantification of human osteoarthritic knee joint effusion volume using magnetic resonance imaging. Arthritis Res. Ther..

[41] Porter A., Newcomb E., DiStefano S., Poplawski J., Kim J., Axe M., Lucas Lu X. (2024). Triamcinolone acetonide has minimal effect on short- and long-term metabolic activities of cartilage. J. Orthop. Res..

[42] Euppayo T., Siengdee P., Buddhachat K., Pradit W., Chomdej S., Ongchai S., Nganvongpanit K. (2016). In Vitro effects of triamcinolone acetonide and in combination with hyaluronan on canine normal and spontaneous osteoarthritis articular cartilage. Vitr. Cell. Dev. Biol. Anim..

[43] Spitzer A.I., Richmond J.C., Kraus V.B., Gomoll A., Jones D.G., Huffman K.M., Peterfy C., Cinar A., Lufkin J., Kelley S.D. (2019). Safety and Efficacy of Repeat Administration of Triamcinolone Acetonide Extended-release in Osteoarthritis of the Knee: A Phase 3b, Open-label Study. Rheumatol. Ther..

[44] Arnold K.M., Sicard D., Tschumperlin D.J., Westendorf J.J. (2023). Atomic Force Microscopy Micro-Indentation Methods for Determining the Elastic Modulus of Murine Articular Cartilage. Sensors.

[45] Sliogeryte K., Botto L., Lee D.A., Knight M.M. (2016). Chondrocyte dedifferentiation increases cell stiffness by strengthening membrane-actin adhesion. Osteoarthr. Cartil..

[46] Song J., Zeng X., Li C., Yin H., Mao S., Ren D. (2024). Alteration in cartilage matrix stiffness as an indicator and modulator of osteoarthritis. Biosci. Rep..

[47] Danalache M., Kleinert R., Schneider J., Erler A.L., Schwitalle M., Riester R., Traub F., Hofmann U.K. (2019). Changes in stiffness and biochemical composition of the pericellular matrix as a function of spatial chondrocyte organisation in osteoarthritic cartilage. Osteoarthr. Cartil..

[48] Schuh E., Kramer J., Rohwedel J., Notbohm H., Müller R., Gutsmann T., Rotter N. (2010). Effect of matrix elasticity on the maintenance of the chondrogenic phenotype. Tissue Eng. Part A.

[49] Trickey W.R., Lee G.M., Guilak F. (2000). Viscoelastic properties of chondrocytes from normal and osteoarthritic human cartilage. J. Orthop. Res..

[50] Fackler N.P., Yareli-Salinas E., Callan K.T., Athanasiou K.A., Wang D. (2023). In Vitro Effects of Triamcinolone and Methylprednisolone on the Viability and Mechanics of Native Articular Cartilage. Am. J. Sports Med..

[51] Danalache M., Jacobi L.F., Schwitalle M., Hofmann U.K. (2019). Assessment of biomechanical properties of the extracellular and pericellular matrix and their interconnection throughout the course of osteoarthritis. J. Biomech..

[52] Wilusz R.E., Zauscher S., Guilak F. (2013). Micromechanical mapping of early osteoarthritic changes in the pericellular matrix of human articular cartilage. Osteoarthr. Cartil..

[53] Gilbert S.J., Bonnet C.S., Blain E.J. (2021). Mechanical Cues: Bidirectional Reciprocity in the Extracellular Matrix Drives Mechano-Signalling in Articular Cartilage. Int. J. Mol. Sci..

[54] Lv M., Zhou Y., Chen X., Han L., Wang L., Lu X.L. (2018). Calcium signaling of in situ chondrocytes in articular cartilage under compressive loading: Roles of calcium sources and cell membrane ion channels. J. Orthop. Res..

[55] Zhang M., Meng N., Wang X., Chen W., Zhang Q. (2022). TRPV4 and PIEZO Channels Mediate the Mechanosensing of Chondrocytes to the Biomechanical Microenvironment. Membranes.

[56] Savadipour A., Nims R.J., Rashidi N., Garcia-Castorena J.M., Tang R., Marushack G.K., Oswald S.J., Liedtke W.B., Guilak F. (2023). Membrane stretch as the mechanism of activation of PIEZO1 ion channels in chondrocytes. Proc. Natl. Acad. Sci. USA.

[57] Zhang Q.Y., Bai J.D., Wu X.A., Liu X.N., Zhang M., Chen W.Y. (2020). Microniche geometry modulates the mechanical properties and calcium signaling of chondrocytes. J. Biomech..

[58] Lopez-Ayon G.M., Oliver D.J., Grutter P.H., Komarova S.V. (2012). Deconvolution of calcium fluorescent indicator signal from AFM cantilever reflection. Microsc. Microanal..

[59] Hsieh C.H., Lin Y.H., Lin S., Tsai-Wu J.J., Herbert Wu C.H., Jiang C.C. (2008). Surface ultrastructure and mechanical property of human chondrocyte revealed by atomic force microscopy. Osteoarthr. Cartil..

[60] Gnanasambandam R., Ghatak C., Yasmann A., Nishizawa K., Sachs F., Ladokhin A.S., Sukharev S.I., Suchyna T.M. (2017). GsMTx4: Mechanism of Inhibiting Mechanosensitive Ion Channels. Biophys. J..

[61] Amin A.K., Huntley J.S., Bush P.G., Simpson A.H., Hall A.C. (2009). Chondrocyte death in mechanically injured articular cartilage--the influence of extracellular calcium. J. Orthop. Res..

[62] Lee J.-S., Im G.-I. (2011). SOX Trio Decrease in the Articular Cartilage with the Advancement of Osteoarthritis. Connect. Tissue Res..

[63] Zhong L., Huang X., Karperien M., Post J.N. (2016). Correlation between Gene Expression and Osteoarthritis Progression in Human. Int. J. Mol. Sci..

[64] Zhang Q., Ji Q., Wang X., Kang L., Fu Y., Yin Y., Li Z., Liu Y., Xu X., Wang Y. (2015). SOX9 is a regulator of ADAMTSs-induced cartilage degeneration at the early stage of human osteoarthritis. Osteoarthr. Cartil..

[65] Tempfer H., Gehwolf R., Lehner C., Wagner A., Mtsariashvili M., Bauer H.C., Resch H., Tauber M. (2009). Effects of crystalline glucocorticoid triamcinolone acetonide on cultered human supraspinatus tendon cells. Acta Orthop..

[66] Kypriotou M., Fossard-Demoor M., Chadjichristos C., Ghayor C., de Crombrugghe B., Pujol J.P., Galéra P. (2003). SOX9 exerts a bifunctional effect on type II collagen gene (COL2A1) expression in chondrocytes depending on the differentiation state. DNA Cell Biol..

[67] Uddin M.N., Siddiq A., Oettinger C.W., D’Souza M.J. (2011). Potentiation of pro-inflammatory cytokine suppression and survival by microencapsulated dexamethasone in the treatment of experimental sepsis. J. Drug Target..

[68] Mukherjee A., Das B. (2024). The role of inflammatory mediators and matrix metalloproteinases (MMPs) in the progression of osteoarthritis. Biomater. Biosyst..

[69] Rübenhagen R., Schüttrumpf J.P., Stürmer K.M., Frosch K.-H. (2012). Interleukin-7 levels in synovial fluid increase with age and MMP-1 levels decrease with progression of osteoarthritis. Acta Orthop..

[70] Burrage P.S., Mix K.S., Brinckerhoff C.E. (2006). Matrix Metalloproteinases: Role in Arthritis. Front. Biosci..

[71] Stefani R.M., Lee A.J., Tan A.R., Halder S.S., Hu Y., Guo X.E., Stoker A.M., Ateshian G.A., Marra K.G., Cook J.L. (2020). Sustained low-dose dexamethasone delivery via a PLGA microsphere-embedded agarose implant for enhanced osteochondral repair. Acta Biomater..

[72] Guilak F., Zell R.A., Erickson G.R., Grande D.A., Rubin C.T., McLeod K.J., Donahue H.J. (1999). Mechanically induced calcium waves in articular chondrocytes are inhibited by gadolinium and amiloride. J. Orthop. Res..

[73] Loewenstein W.R., Rose B. (1978). Calcium in (junctional) intercellular communication and a thought on its behavior in intracellular communication. Ann. N. Y. Acad. Sci..

[74] He F., Wang L., Umrath F., Naros A., Reinert S., Alexander D. (2024). Three-Dimensionally Cultured Jaw Periosteal Cells Attenuate Macrophage Activation of CD4+ T Cells and Inhibit Osteoclastogenesis. Int. J. Mol. Sci..

[75] Umrath F., Thomalla C., Pöschel S., Schenke-Layland K., Reinert S., Alexander D. (2018). Comparative Study of MSCA-1 and CD146 Isolated Periosteal Cell Subpopulations. Cell Physiol. Biochem..

[76] Livak K.J., Schmittgen T.D. (2001). Analysis of relative gene expression data using real-time quantitative PCR and the 2^−ΔΔCT^ Method. Methods.

